# Mermaid Syndrome: A Case Report in Mauritius

**DOI:** 10.7759/cureus.2210

**Published:** 2018-02-20

**Authors:** Kamleshun Ramphul, Stephanie G Mejias, Yogeshwaree Ramphul-Sicharam

**Affiliations:** 1 Department of Pediatrics, Shanghai Xin Hua Hospital Affiliated to Shanghai Jiao Tong University School of Medicine, Shanghai, People's Republic of China; 2 Department of Pediatrics, Robert Reid Cabral Children's Hospital Affiliated to the University Iberoamericana Unibe School of Medicine; 3 Sir Seewoosagur Ramgoolam National Hospital

**Keywords:** sirenomelia, mermaid syndrome, caudal regression syndrome

## Abstract

Sirenomelia is a rare congenital malformation that results in the fusion of the lower limbs together with multiple visceral anomalies. We report a case of sirenomelia observed in Mauritius and the different findings seen in the baby. The baby had fused lower extremities and bony structures for each leg were present. The umbilical cord consisted of a single artery and one vein. The external genitalia was absent and an imperforate anus was also seen. An x-ray revealed poorly expanded lungs and two distinct sets of femur and tibia were seen on imaging. However, a fused fibula and a fused talus were also noted. Multiple theories have been suggested for the pathogenesis of this condition, and despite recent progress in pathology, this condition remains debated.

## Introduction

Mermaid syndrome, also known as sirenomelia, is a rare congenital malformation that is often fatal [1]. The incidence of mermaid syndrome is estimated to be 0.8 - 4 in 100,000 pregnancies [2] and is spread over different ethnic groups and communities around the world. Males and monozygotic twins are more at risk for this condition.

The most common feature seen in sirenomelia is the complete or partial fusion of the lower limbs into a single lower limb, giving it a mermaid resemblance. There are usually multiple underlying visceral abnormalities that make it incompatible with life with a few rare exceptions of infants surviving with this condition.

We report a case of sirenomelia seen in Mauritius and the associated findings.

## Case presentation

A 23-year-old female G1P0 was admitted for an elective Cesarean section at week 36. The pregnancy was from a non-consanguineous union with a 25-year-old male. She was not compliant with her antenatal consultations and a late diagnosis of possible sirenomelia was made when she had her first ultrasound on admission. The few blood tests done before admission showed no anomalies. Her pregnancy was uneventful, and she was not exposed to any teratogenic drugs or illnesses.

A 1,800 g baby was extracted during the Cesarean section. The baby did not cry at birth and the APGAR scores were four at one minute, five at five minutes. Proper measures were provided to keep the baby alive, but after 15 minutes of birth, the baby was declared dead.

**Figure 1:**
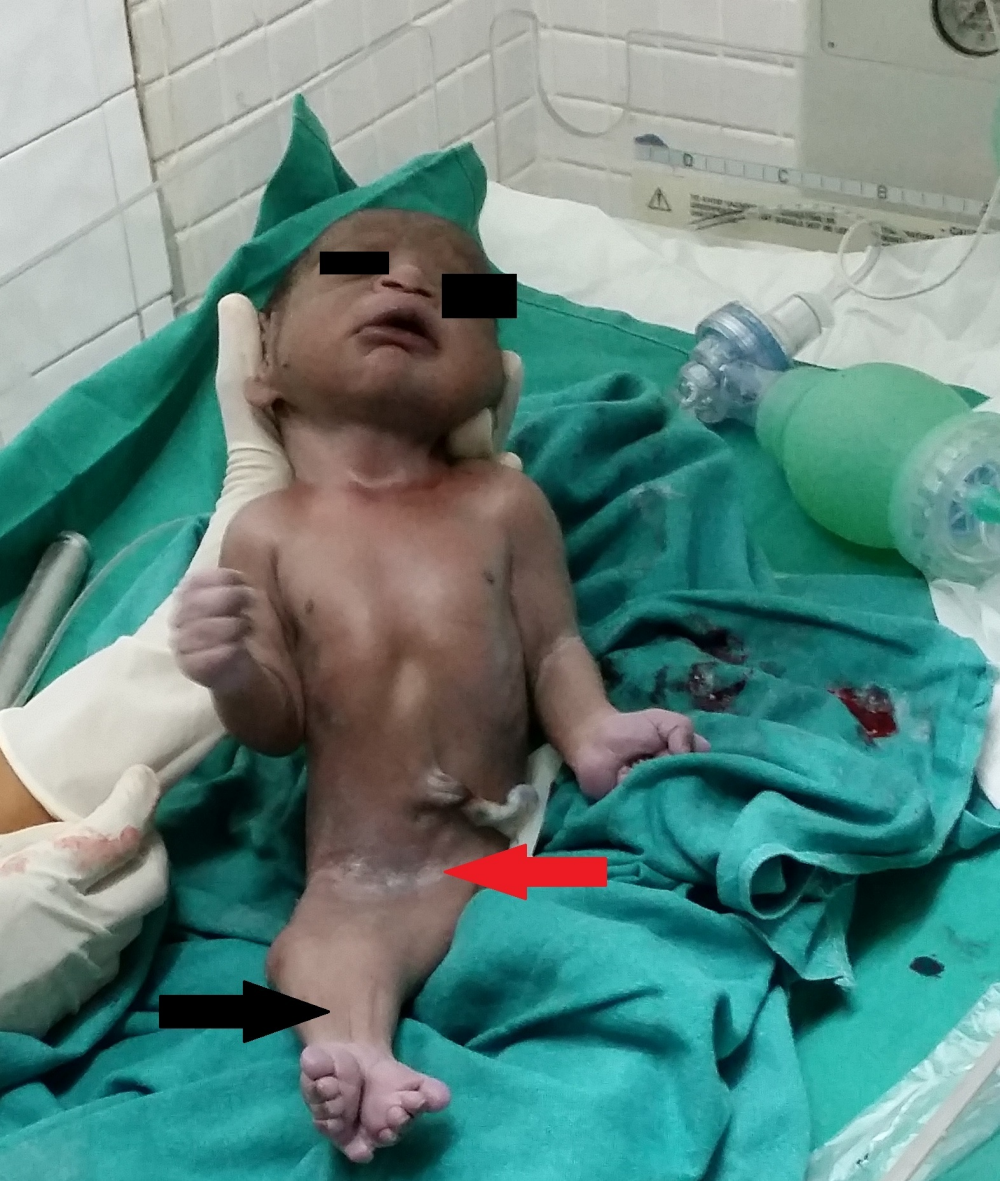
Newborn with sirenomelia Absent external genitalia (red arrow) and fused lower extremities (black arrow) were seen in the newborn.

**Figure 2:**
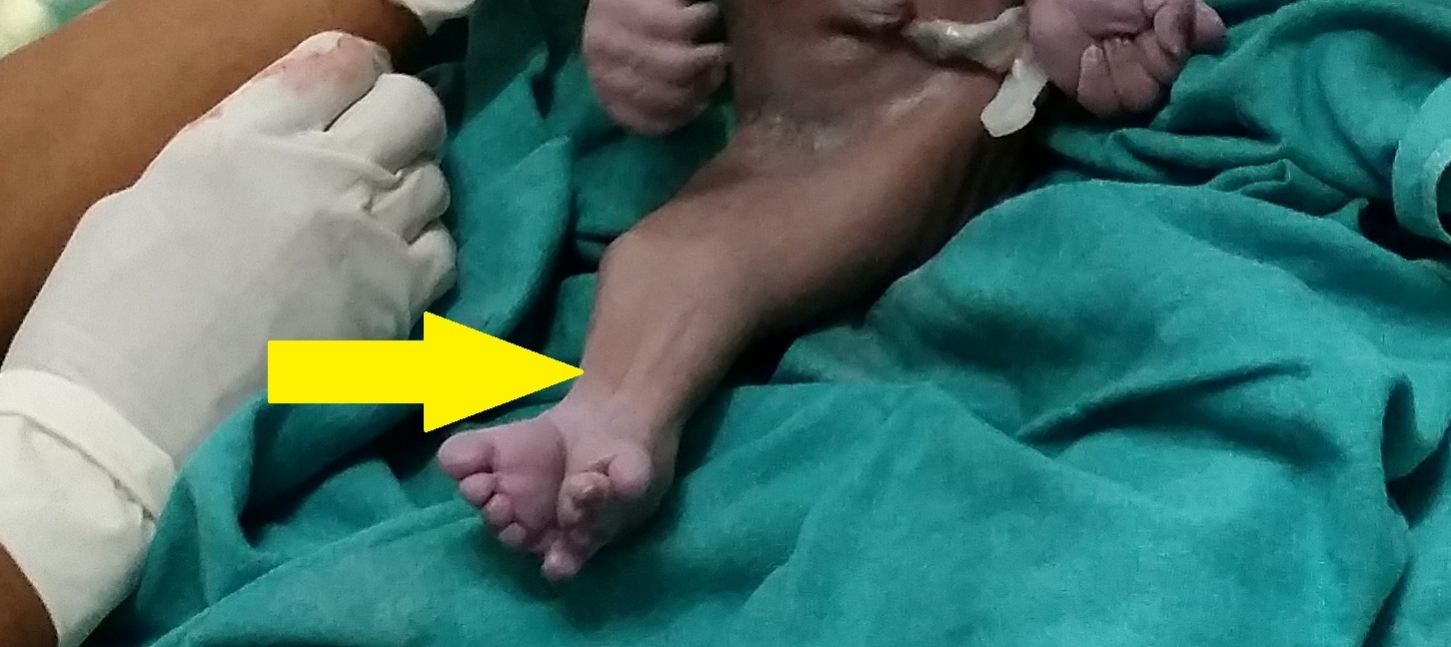
Distal end of lower limb fused in sirenomelia newborn Completely fused lower extremities (yellow arrow) on examination and two palpable sets of femur and tibia.

**Figure 3:**
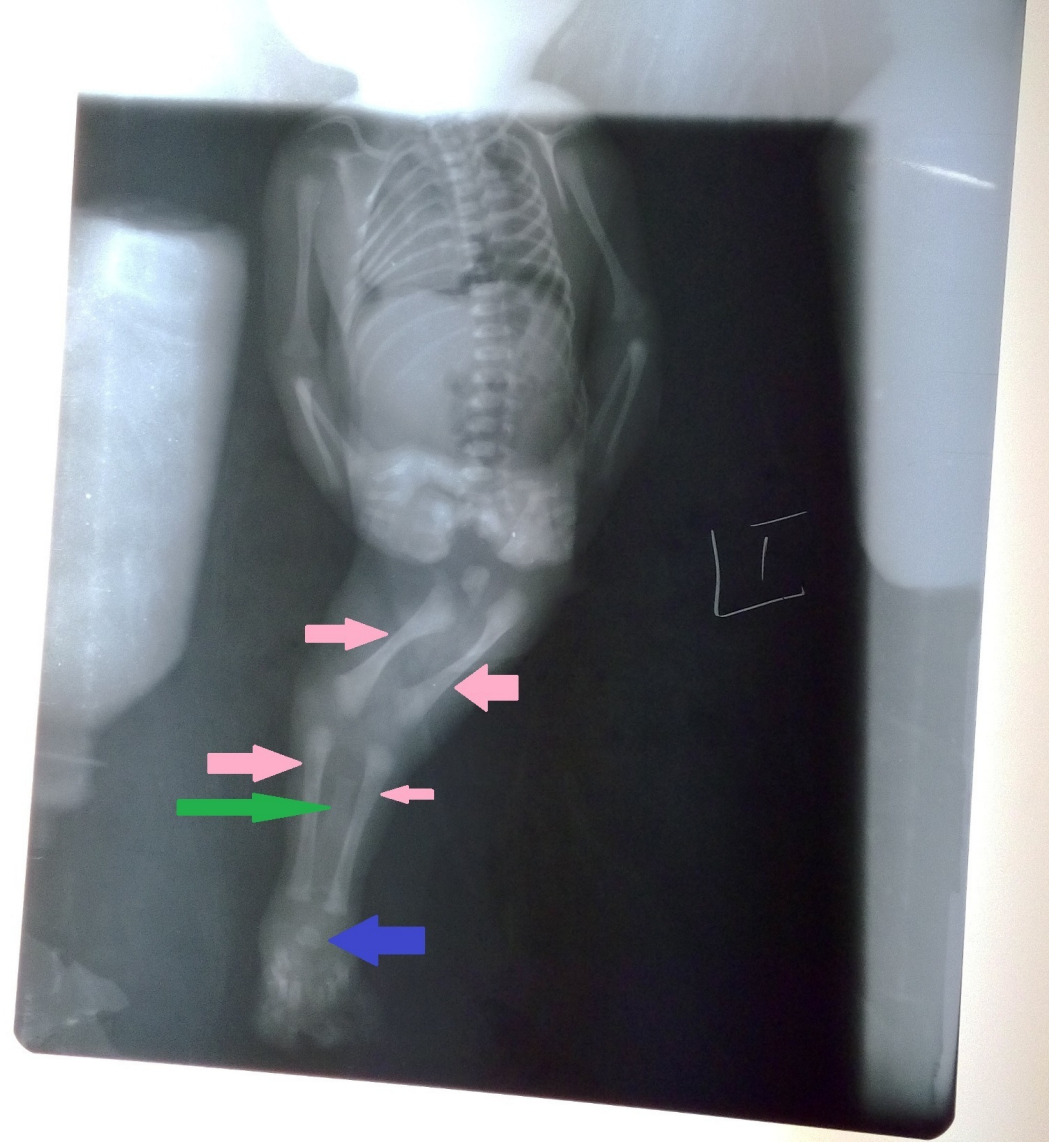
X-ray of newborn with sirenomelia Two different sets of femur and tibia were present (pink arrows); however, a fused fibula (green arrow) and fused talus (blue arrow) were also noted.

On physical examination, the baby had fused legs and bony structures for each leg were palpated (Figures 1, 2). The umbilical cord consisted of a single artery and one vein. The external genitalia was absent in the baby and an imperforate anus was also observed. An x-ray revealed poorly expanded lungs, and two distinct sets of femur and tibia were seen on imaging. However, a fused fibula and a fused talus were also noted (Figure 3).

## Discussion

Mermaid syndrome involves an abnormal development of the caudal region resulting in varying degrees of fusion of the lower limbs with or without bony defects. There are multiple associated visceral defects that can be present, such as cardiac agenesis, hypoplastic lungs, absent genitalia, and imperforate anus. Recent studies have shown that there can be a genetic factor predisposing to the development of sirenomelia. Death usually results from hypoplastic lungs or from renal failure. The parents did not consent for an autopsy, and we were unable to determine the presence of any renal anomalies.

Maternal diabetes is a major risk factor for multiple caudal anomalies during pregnancy [3]. However, only 0.5 - 3.7% of sirenomelia cases have been reported in diabetic mothers [4]. The etiopathogenesis has been debatable and multiple theories have been proposed. Stevenson et al. suggested that there is a shunting of blood via an abnormal abdominal artery that leaves the caudal end of the embryo poorly perfused, causing a vascular steal condition [5]. This leads to a poorly perfused caudal part that undergoes complete or incomplete agenesis of the caudal structures. A possible vertebral dysgenesis leading to lower limb atrophy and inconsistent lower limb fusion can also be present [6]. A case of sirenomelia without any arterial steal was published by Jaiyessimi et al. suggesting that there are other factors that could also be involved in the pathogenesis [7]. The presence of teratogens, such as retinoic acid, cyclophosphamide, and cadmium, has been reported in the genesis of sirenomelia in animal studies [8-9]. Several genetic factors leading to caudal regression syndrome that could be associated with sirenomelia have also been reported. There could be a multifactor polygenetic transmission, dominant sex-linked transmission, and dominant autosomal transmission with a variable expression and an attenuated penetrance [10].

Mermaid syndrome is fatal in most cases due to pulmonary hypoplasia and renal failure resulting from renal agenesis. Half of the children with sirenomelia are born alive and most die within the next five days. Very few cases have been reported where a child with sirenomelia survived. The management of the complications associated with this condition proved to be costly and difficult, and the quality of life in most of the survivals is considered debatable.

## Conclusions

Mermaid syndrome is a rare condition with a poor prognosis. There are multiple controversies on the pathogenesis and the conditions predisposing to this condition. Antenatal diagnosis is possible via ultrasound, albeit difficult. The prognosis, quality of life, and associated complications of survivors have also divided the scientific community. More emphasis should be laid on proper prenatal diagnosis and care with a possible termination of pregnancy proposed as an option if detected early.
